
JOURNAL INFORMATION
==============================
NLM Title Abbreviation: ScientificWorldJournal
Journal ID: ScientificWorldJournal
NLM Journal ID: 101131163
Journal ID: TSWJ

The Scientific World Journal

ISSN: 2356-6140
EISSN: 1537-744X
Publisher: Wiley

ARTICLE INFORMATION
==============================
PMCID: PMC6009255
PMID: 29973802
DOI: 10.1100/tsw.2002.28
Article ID: 320285
Article version: 1
Subjects: Short Report

Biochemical and Structural Analysis of the Role of the Wlb Gene Locus in Bordetella Pertussis Lipopolysaccharide Biosynthesis

Kannathasan Velupillai S. 1
Dong Chang J. 2
Wing Corin 1
Preston Andrew 3
Maskell Duncan J. 3
Naismith James H. 2
Field Robert A. 1 *
Centre for Carbohydrate Chemistry, University of East Anglia, Norwich, NR4 7TJ, UK
Centre for Biomolecular Sciences, University of St Andrews, KY16 9ST, UK
School of Veterinary Medicine, University of Cambridge, Cambridge, CB3 OES, UK
*Robert A. Field: r.a.field@uea.ac.uk
Publication date: 2002
Electronic publication date: 2002 Apr 20
Volume: 2
First page: 55
Last page: 56
Copyright: Copyright © 2002 Velupillai S. Kannathasan et al.
Copyright year: 2002
License: This is an open access article distributed under the Creative Commons Attribution License, which permits unrestricted use, distribution, and reproduction in any medium, provided the original work is properly cited.
License URL: https://creativecommons.org/licenses/by/3.0/

Funding: Biotechnology and Biological Sciences Research Council

==============================
Advances in Gene Technology: The Genome and Beyond –
Structural Biology for Medicine (Proceedings of the 2002 Miami
*Nature Biotechnology* Winter Symposium)
*TheScientificWorld* 2002, 2(S2), 55–56
ISSN 1532-2246; DOI 10.1100/tsw.2002.28

### BIOCHEMICAL AND STRUCTURAL ANALYSIS OF THE ROLE OF THE *WLB* GENE LOCUS IN *BORDETELLA PERTUSSIS* LIPOPOLYSACCHARIDE BIOSYNTHESIS

Velupillai S. Kannathasan[a], Chang J. Dong[b], Corin Wing[a], Andrew Preston[c], Duncan J. Maskell[c], James H. Naismith[b], and Robert A. Field[a,]*
[a]Centre for Carbohydrate Chemistry, University of East Anglia, Norwich, NR4 7TJ, U.K.; [b]Centre for Biomolecular Sciences, University of St Andrews, KY16 9ST, U.K.; [c]School of Veterinary Medicine, University of Cambridge, Cambridge, CB3 OES, U.K.
*r.a.field@uea.ac.uk

**INTRODUCTION.** *Bordetella pertussis* is the causative agent of whooping cough in children and chronic cough in adults. *Bordetella* lipopolysaccharide (LPS) has been implicated in the infection process and has been shown to be highly immunogenic, and acts as an immunological adjuvant which displays the properties of an endotoxin. Tentative gene function assignments have been made for the *B. pertussis* LPS band A trisaccharide biosynthetic locus (*wlb*), albeit based on rather weak sequence similarities[1,2]. The *B. pertussis wlbD* gene product is a putative uridine-5-diphosphate N-acetylglucosamine 2'-epimerase based on its homology to *E. coli rffE* (32% identical), an established UDP-GlcNAc 2'-epimerase that is involved in Enterobacterial Common Antigen (ECA) formation. The key active site residues in *rffE* are present in the *wlbD* sequence.

**RESULTS AND DISCUSSION**. The *wlbD* gene from *B. pertussis* has been cloned and over-expressed in *E. coli* and the resulting protein has been purified to homogeneity. All the activity assays failed to show the epimerase activity *in vitro* and *wlbD* failed to complement the *E. coli rffE* mutant. This suggests that assignment of *wlbD* is almost certainly wrong, which prompts re-examination of the entire biosynthetic pathway (see Fig.1).The most likely substrate for *wlbD* may be UDP-Glc-2,3-diNAc. The 3-D structure of *wlbD* may throw light on its function, crystals of the mutant Gln339Arg *wlbD* enzyme have been obtained in the presence and absence of UDP-GlcNAc and 1.6 Å (in Grenoble, France) and 2.3 Å (in house) data sets were collected, respectively.[4] In order to solve the structure a Seleno-methionine variant protein was prepared and MAD data were collected since molecular replacement using *rffE* as model failed. The crystal structure exists as a dimer and each monomer has two domains (see Fig. 2). The *wlbD* structure shares marked topological similarity to the *rffE* structure and other glycosyltransferases (DALI analysis). Further structural analysis is going on.

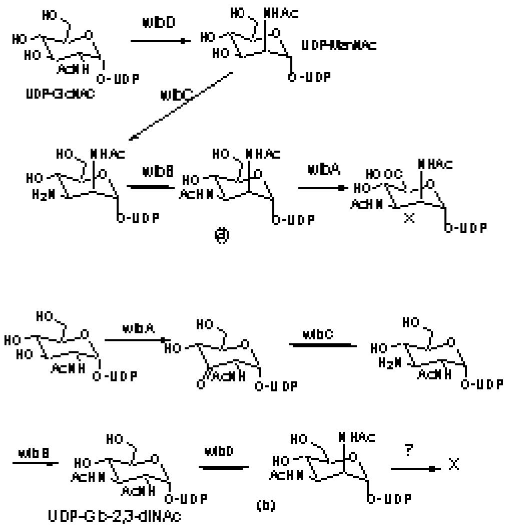

**FIGURE 1**. (a) Original proposed pathway; (b) revised pathway.

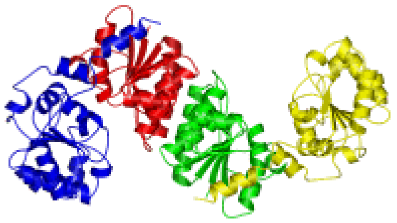

**FIGURE 2**. Crystal structure of *wlbD.*

**ACKNOWLEDGEMENT.** This work was supported by the UK Biotechnology and Biological Sciences Research Council.

**REFERENCES**

1. Allen, A.G. and Maskell, D.J. (1996) Mol. Microbiol. 19, 37–52.
2. Allen, A.G., Thomas, R.M., Cadisch, J.T., and Maskell, D.J. (1998) Mol. Microbiol. 29, 27–38.
3. Campbell, R.E., Mosimann, S.C., Tanner, M.E., and Strynadka, N.C.J. (2000) Biochemistry 49, 14993–15001.
4. Kannathasan, V.S., Staines, A.G., Dong, C.J., Preston, A.G., Maskell, D.J., Naismith, J.H., and Field, R.A. (2001) Acta Crystallogr. D57, 1310–1312.
